# Measuring Key Quality Indicators in Cloud Gaming: Framework and Assessment Over Wireless Networks

**DOI:** 10.3390/s21041387

**Published:** 2021-02-17

**Authors:** Oswaldo Sebastian Peñaherrera-Pulla, Carlos Baena, Sergio Fortes, Eduardo Baena, Raquel Barco

**Affiliations:** Departamento de Ingeniería de Comunicaciones, Campus de Teatinos s/n, Universidad de Málaga, Andalucía Tech, 29071 Málaga, Spain; sppulla@ic.uma.es (O.S.P.-P.); jcbg@ic.uma.es (C.B.); ebm@ic.uma.es (E.B.); rbm@ic.uma.es (R.B.)

**Keywords:** cloud gaming, quality of experience, key quality indicators, service performance, wireless networks, service architecture

## Abstract

Cloud Gaming is a cutting-edge paradigm in the video game provision where the graphics rendering and logic are computed in the cloud. This allows a user’s thin client systems with much more limited capabilities to offer a comparable experience with traditional local and online gaming but using reduced hardware requirements. In contrast, this approach stresses the communication networks between the client and the cloud. In this context, it is necessary to know how to configure the network in order to provide service with the best quality. To that end, the present work defines a novel framework for Cloud Gaming performance evaluation. This system is implemented in a real testbed and evaluates the Cloud Gaming approach for different transport networks (Ethernet, WiFi, and LTE (Long Term Evolution)) and scenarios, automating the acquisition of the gaming metrics. From this, the impact on the overall gaming experience is analyzed identifying the main parameters involved in its performance. Hence, the future lines for Cloud Gaming QoE-based (Quality of Experience) optimization are established, this way being of configuration, a trendy paradigm in the new-generation networks, such as 4G and 5G (Fourth and Fifth Generation of Mobile Networks).

## 1. Introduction

In recent years, most services are evolving to focus on providing new features and experiences to their final users. In this context, the gaming industry has been one of the top essential fields in the technology businesses because of the great profits of the sector (152.1 billion dollars projected for 2019) [1]. The gaming platforms also have changed along the time, starting from large consoles in the 80s and 90s to software that can be hosted in dedicated personal computers [2].

Hardware advances have also supported the application of a great variety of computer graphic techniques, which has currently led to the creation of very realistic video games, allowing the player to immerse in a virtual world. In this context, gaming products are commonly used in the entertainment field; however, this is not the only use of this service. This fact has promoted employing gaming products beyond entertainment, being increasingly common the use of simulators with the aim of training people to develop all the aptitudes that could be obtained in the real field [3]. Nevertheless, this entails heavy computational processes in order to render high-quality scenes, which means a large investment in equipment, such as powerful graphic cards and memories.

Initially, video games were designed to depict a virtual 2D (two-dimensional) environment through a monitor. The graphic processing was delegated to a piece of specific equipment known as console. In this context, the very first gaming methodology was called “Arcade”, where the user could interact with the game through several missions or stories in its own way. Over the years, gaming providers presented a new way to interact within a game with several players simultaneously, called multiplayer games, where users traditionally need to stay in the same place as the console. Nonetheless, this restriction has been overcome since this approach takes advantage of the current intercommunication facilities through the Internet. In this way, the game logic is now moved from the console to an online server, which is in charge of updating all users with the session status. However, this does not avoid that users have to run the gaming application on its side, which sometimes obliges users to invest in equipment. Besides, the lack of adequate gaming equipment highly affects the user experience, which is one of the most important shortcomings that the sector always has to overcome.

Cloud Gaming is a new approach that looks for reducing the computational processing effort in the client-side, thus delegating several tasks to the server-side. The server acts on behalf of the client replicating user actions and game logic with the external online gaming server located somewhere on the Internet [4,5]. In this way, some companies, such as Google or Nvidia, have begun to provide Gaming as a Service (GaaS) with their platforms Stadia and GeForceNow respectively. This kind of solutions try to offer a complete gaming solution featured for a group of ready-to-play applications. Conversely, these demand more resources from the connection between the client and the server. Due to the need of sending multimedia data which has been rendered in the server, a user’s connection must be able to send a large amount of data that conforms these content with the lowest possible latency, like in online gaming [6]. Hence, this kind of services are more network sensitive, which requires to assure a set of specific requirements that allow providing the best possible Quality of Experience (QoE) [4,7].

As far as the authors’ knowledge, the bibliographic research shows that there are no works that actually present an integral analysis on the impact of session configuration over the service quality in Cloud Gaming. Besides, there is no reference of implementations of Cloud Gaming service using different technologies in the transport network. Neither was found some paper that presents statistical studies using session configuration and Key Quality Indicators (KQIs), in order to found out relationships in service performance and quality of experience.

The work developed by Beyer et al. [8] presents an implementation of this service using the Stream-a-Game platform. In the same context, Slivar et al. [9] analyzes the performance of GamingAnywhere (GA) platform over a WLAN (Wireless Local Area Network) for educational purposes using subjective criteria from end-user. That paper measures the QoE using traditional MOS (Mean Opinion Score) techniques as surveys gathered from gaming users. Another topic covered in previous studies is the impact of resource mobilization to the cloud and how this change can improve user experience. In Reference [10], a mobile Cloud Gaming service is provided using light data centers called cloudlet. This paper shows the impact of using distributed infrastructure that absorbs rendering tasks; hence, mobile terminals are just used to capture user actions and display video. The results show the system performance and the distribution of latencies in several Android terminals. Lyn et al. [11] presents an implementation using supernodes in the fog to reduce latency by distributing processing tasks to a server close to the user. However, cloud central servers are conserved in order to manage the system. Continuing this study, the authors in Reference [12], conduct simulations using this architecture for MMOG (Massive Multimedia Online Games) with high QoE. This paper analyzes encoding rate as a factor for latency reduction and, hence, an increase in the service experienced quality.

Moreover, research has been done to analyze the impact of specific parameters in the QoE of this service. Sabet et al. [13] shows a subjective study of delay sensitivity, which depends on the applied strategy in game. The research performed by Semsarzadeh et al. [14] is focused on how encoding optimization can be made in order to reduce process complexity and reduce and speed up the video delivery. Meanwhile, References [15,16] propose algorithms that are able to establish the importance of each frame in the impact of the game experience. If a frame is quite relevant, it is encoded with a high bitrate. The reversal situation occurs with non-relevant frames. It allows to reduce the needed bitrate in the session and improving the service for bounded link speeds.

Regarding resource allocation, Cloud Gaming is a service that needs to find out a trade-off between server performance and the quality of experience. In Reference [17], a framework is developed to manage virtual processing and network resources allocation in order to maintain high QoE with the lowest processing load on the server side. Conversely, Reference [18] presents a price-based approach, which is grouped into different categories in function of image quality rendering, to dynamically adapt session settings to get the best cloud performance without affecting the user experience and its preferences.

Nevertheless, there is no previous work that studies how to measure QoE or determine network impact for the Cloud Gaming service as it exists for other services. Previous publications refer recommend KQIs in order to assess the QoE of different services [19]. Other works, such as Herrera-Garcia et al. [20], present a KQI modeling Artificial Intelligence (AI)-based strategy for video streaming and FTP services, which enables a QoE-based network management in a real cellular network [21]. In the same context, the authors in Reference [22] introduce an application of KQI modeling for network slicing in video streaming, where KQIs are estimated using AI-based methods from application data, giving a provider-network operator perspective. Other studies for QoE indicators are made for LTE video streaming and Voice over LTE (VoLTE) [23]. This paper depicts subjective QoE using mathematical models that weigh several key performance indicators (KPIs) impact over the KQIs. Meanwhile, Krogfross et al. [24] describes a theoretical methodology to estimate QoE using throughput and latency in Augmented Reality/Virtual Reality (AR/VR) services in 5G through the Edge computing paradigm.

Nonetheless, due to its recent expansion, there are no works that analyze the impact of the network on Cloud Gaming KQIs, which enables a service-oriented network configuration as proposed in References [20,22].

To overcome the identified shortcomings of the bibliography, the present paper establishes a baseline for objective measurement to assess Cloud Gaming performance in terms of network impact and multimedia configuration.

In this work, the design, implementation, and testing of a novel extraction framework for Cloud Gaming are described. This framework allows assessing the impact generated by some session parameters in the service provision in terms of latency and frame losses. Moreover, this framework eases the evaluation of the Cloud Gaming performance through its different KQIs over different network technologies, such as Ethernet, WiFi, and LTE.

This assessment is performed via a set of automated tests, in which a user is emulated interacting with the service through different combinations of video resolution and frame rate. In this way, this work establishes a qualitative synthesis on how each technology develops the service, providing a clear vision on how the performance of this kind of service is mostly impacted by the network.

Finally, an overall KQI-service configuration relation synthesis for LTE is done using correlation methods in order to establish the key points where optimization might be performed in future works.

This paper is organized as follows. First, Section 2 presents an overview of the Cloud Gaming service in comparison with traditional online gaming and key points of the selected platform for this developed work. Then, Section 3 explains the system architecture that consists of a server-client model and two software tools used for actions automation and control and indicators extraction.

Following that, Section 4 shows the key indicators obtained by several tests that were made using the Cloud Gaming service over the different technologies, such as LTE, WiFi, and Ethernet, as the transport network. Besides, a statistical study is performed in order to determine a correlation between configuration parameters and KQIs. These results demonstrate, in a quantitative way, the network impact over the Cloud Gaming service.

Finally, Section 5 presents the conclusions that show the key points that summarize the impact of multimedia configuration, as well as the transport network over the KQIs in Cloud Gaming service.

## 2. Cloud Gaming Overview

Cloud Gaming is a new philosophy that looks for executing gaming software with high graphic processing requirements in a remote way through a server located in the cloud. With this premise, the client just needs to have a high-speed Internet connection to receive the multimedia data of the game (video and audio) and send information that includes user commands and session control data. This new scope allows the user side to be simple in terms of non-dedicated hardware equipment and bringing all the hard processing tasks to the cloud [4,25]. This section presents a comparison between online and Cloud Gaming, followed for an overview of various platforms that are used in this service. To end this section, a QoE description for Cloud Gaming is introduced, which considers the key factors that impact over the experience.

### 2.1. Online and Cloud Gaming Comparison

**Figure 1:**
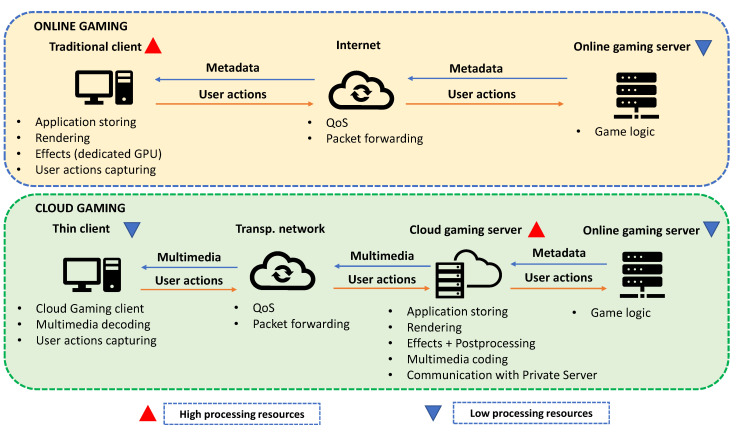
Online and Cloud Gaming features.

The Cloud Gaming structure can be differentiated from the “traditional” online gaming paradigm. The first one assures some advantages in terms of computational equipment, however, requires to manage some network and processing parameters in order to guarantee an adequate QoE. Figure 1 presents the key instances and tasks in both cases [26].

The online gaming approach is featured by two key elements: the online gaming server, most cases owned by the game provider, and the client, which is used by the final user. The first one is in charge of managing the game logic from user actions sent by the clients. On the other side, the client has to perform storage, graphic processing and user actions capturing tasks. These functions need high-processing-enabled hardware, such as GPU (Graphics Processing Unit) and CPU (Central Processing Unit), in order to assure the best user QoE. However, the data exchange between these two instances is based on application data, which means low traffic to be delivered with high reliability and low latency [26,27].

Meanwhile, Cloud Gaming key point is to reduce the high processing requirements in the client side. To reach this goal several tasks are delegated to an intermediate element known as “cloud gaming server”. This server is in charge of game storing, graphic processing (rendering and effects), and multimedia encoding.

Considering this scenario, the client requirements are reduced in comparison with a traditional online gaming client. This fact, let this element be called a “thin client” [7,26]. Nevertheless, the online gaming case is not directly applicable to the Cloud Gaming approach. If two players are located geographically at two different points, the Cloud Gaming architecture must assure that each user connects to the closest server, say a CDN-based (Content Delivery Network) approach, to guarantee the lowest latency possible. Then, the interconnection between Cloud Gaming servers, which are acting on behalf of the user, might be considered an online gaming problem.

Furthermore, the cloud approach is based on the transport of multimedia streams from the server to the thin client, which means high traffic that should be delivered in real-time in downlink; meanwhile, in uplink, the captured user actions in the thin client are transported to the server. In this way, apart from low latency and high reliability, this paradigm will also require high throughput from the network in order to assure proper QoE. This latter involves the need to know the impact and configuration of the network, which interconnects the Cloud Gaming server and the thin client, in the provision of this service [7,26].

### 2.2. Cloud Gaming Platforms

Different platforms have been developed in order to provide gaming sessions remotely, which are optimized to look for the same experience as in a traditional gaming session. In this context, the Cloud Gaming service could be provided for a pay-per-play or an open-source paradigm, which means to offer to end-users the possibility to pay for an integrated and ready-to-play service or to implement the Cloud Gaming architecture on their own, but free of charge. On the one hand, the first option is implemented via well-known paid platforms, examples of this category are Google Stadia [28] and GeForce Now [29]. On the other hand, on the open-source side, the most known platforms are Gaming Anywhere [30], Rainway [31] and Moonlight-Stream [32].

Conversely to proprietary ones, the open-source platforms allow developers to keep complete control in the creation of Cloud Gaming sessions and the way to implement a server-client architecture without fees. In this case, there is a large number of platforms that could be used for this purpose. Most open-source platforms allow researchers to establish a server-client architecture in order to control and manage Cloud Gaming service. Besides, each one of these platforms uses different transport protocols to stream the multimedia data. In addition, RTP (Real-time Transport Protocol) is considered the most used protocol; however, WebRTC (Web Real Time Communications) is a new interesting choice because of the capability to implement multimedia services directly over web browsers instead of specific software clients. In the near future, this option could propose better compatible solutions that are able to run Cloud Gaming over different operating systems and for a variety of devices.

According to the gathered information, Gaming Anywhere is an integral development project used for the first researching projects in this field, nevertheless, this platform is currently considered discontinued. In this context, Moonlight-Stream and Rainway are leading the open-source projects. These platforms offer multi-platform (host operating system) compatibility. This fact assures quick integration (configuration and video game implementation) through graphical interfaces and search engines. Another key feature of these platforms is the collaborative client improvement, which allows users and researchers around the world to communicate possible new features or found bugs through community chat servers.

A brief comparison between GamingAnywhere [30], Rainway [31], and Moonlight-Stream [32] platforms can be found in Table 1.

In the present work, the Moonlight-Stream platform [32] has been used to develop all the tests. This client presents the best features in comparison with the other two options in terms of availability and infrastructure. Even though Rainway is an open-source platform, only the server is available to be installed in a computer because the remote client is just accessible for mobile operating systems for smartphones, tablets, and smart TVs and web browsers. Meanwhile, as was previously described, Gaming Anywhere currently is outdated. Furthermore, the configuration of the session is simpler, which favors the development of the testbed presented in Section 3.

The Moonlight-Stream Cloud Gaming client is constantly updated and maintains a permanent user-developer communication channel through Discord [33]. This is considered a strength because allows researchers and gaming enthusiasts to develop use cases for the Cloud Gaming service. However, the most important point that supports this choice, is the use of *GameStream* Nvidia proprietary protocol, which is a high-recognized gaming solution and equipment company.

This protocol allows the streaming of video and audio from a server to a *Shield* device from Nvidia. In this context, this platform is able to use *GameStream* data to implement a Cloud Gaming service in an open-source fashion. The key requirement to establish this service is that the server must have a Nvidia dedicated graphics card and its manager *GeForce Experience*. Furthermore, the Moonlight-Stream client is available for multiple operating systems, such as Windows, macOS, Linux, Android, and so on [32].

At the working level, Moonlight Stream use RTP to transport the multimedia stream to the client and the user data to the server. Moreover, the video games can be implemented in the server and the Nvidia manager index them into the service. One of the advantages of this platform is its availability for measuring service metrics [32]. Moonlight does not use adaptive video, so its client needs to configure frame rate and resolution before a session is created, this is due to the use of RTP. Although, it is possible to take advantage of this feature to generate several tests with a fixed configuration in order to analyze Cloud Gaming performance under specific conditions.

### 2.3. Cloud Gaming QoE

The quality of service is traditionally measured using MOS (Mean Opinion Score) methods from subjective measurements. However, objective strategies can be developed through certain variables that are called Key Quality Indicators [34]. These KQIs are statistical values that represent the service performance. In this context, operators have limitations in the KQI gathering due to the information which is protected at the lowest layers. As a consequence, there is the need to extract them from application data at the service level. KQIs allow quantifying the service quality of experience using objective information instead of subjective data from MOS. Moreover, these indicators reduce time and costs that are necessary for traditional QoE assessment. A previous use case for KQIs can be found in References [20,22], where these indicators were estimated and used for decision making in FTP and video streaming services.

In the Cloud Gaming context, the user experience with the service can be harmed due to latencies, packet losses, poor video quality, or lack of smoothness and disconnections. These services features can be quantified through various specific measurements as frame rates, bit rates, network latencies, and packet or frames losses. Besides, the service configuration parameters, such as codecs, video resolution, and bandwidth, have influence over these measurements and, hence, over the quality of the service [35].

Previous works [36,37,38], showed that the latency must be lower than the theoretical limit before degrading the user experience. The latency produces late packet arrival, hence affecting user actions delivery to the server or video frames to the thin client. Several experiments demonstrated that a 100-ms delay is the maximum admissible value for a gaming session.

The work developed by Vlahovic et al., in Reference [36], conducted a study to measure subjective QoE in gaming. The results showed that a 100 ms threshold is needed to provide an adequate gaming environment. When experimented latencies rise from this value, the willingness to keep playing was reduced and the user’s ability to interact with the game was harmed.

In the same context, Beigbeder et al. [39] presented a study generated from participants in a real gaming tournament. The authors conducted a subjective QoE assessment and a statistical study. The results concluded that 100 ms latencies can be noticed by users, and their performance was harmed steadily as this value increases.

When users are using real-time games, their experience can be degraded stronger than in a single player online game. The experiments in Reference [36] show that users are willing to play with a 200 ms latency as a limit. For higher values, users are not willing to play due to experience degradation.

The video quality depends on the resolution and frame rate. A trade-off is needed between these both elements because of the video smoothness. In Reference [40], it is pointed out that an increase in frame rate or resolution produces higher throughputs; hence, there is the necessity of better mechanisms to transport more information in the network. In that work, the tests show that is possible to fix the frame rate for a reduced bitrate capacity. This configuration produces experience degradation due to bottleneck. In another study case, the frame rate is set variable in a bounded high bitrate system. In this scenario, rate changes are irrelevant for the user while its value is not lower than 25 FPS (Frames per Second); therefore, an adequate level of QoE is reached.

Moreover, the packet losses must be bounded in order to assure the required responsiveness and reduce the effect of *input lag*. This problem might produce critical information losses and completely degrade the gaming session and user experience for the Cloud Gaming service. When packets are lost in the uplink, the gaming experience is affected due to the lack of user actions delivery to the server. This effect produces delays in the game responsiveness which is represented for the time the client made an action and the time the action is represented on its screen. The other case is generated when the packets are lost in downlink. In this scenario, video quality is degraded due to frames losses that produce video tearing or discontinuity [9,41].

Summarizing these facts, the Cloud Gaming service requires bounded latencies and packet losses to assure proper responsiveness. In the same context, the network must be able to handle the traffic from an adequate video frame rate and resolution in order to avoid video tearing. The present work develops a testbed to quantify all these metrics in a real Cloud Gaming platform over several technologies in transport network in Section 3. The gathered results are analyzed to establish the impact of session configuration and network in measured KQIs in Section 4.

## 3. Proposed System

In order to develop the system to obtain KQIs from the Cloud Gaming sessions, it is necessary to set up the proposed evaluation framework and its implementation. This comprises hardware and software elements that work simultaneously. The hardware side provides the required equipment to develop a real Cloud Gaming service over an open-source platform. The software side is designed to provide control and automation. This section describes the architecture and software tools of the proposed system.

### 3.1. Framework Architecture

**Figure 2:**
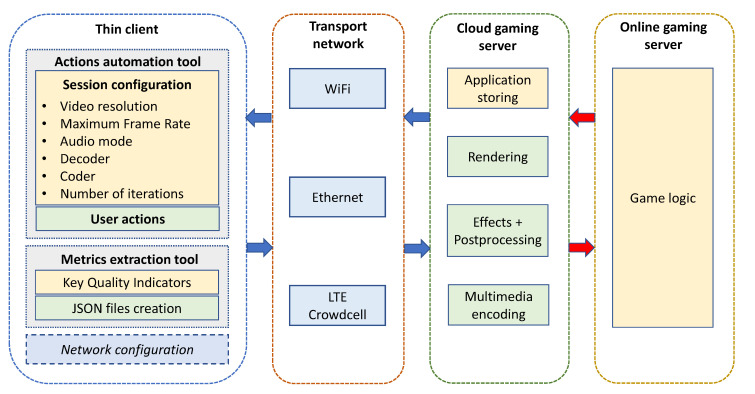
Cloud Gaming system architecture.

The Cloud Gaming system is implemented following a server-thin client architecture. Over the latter are implemented two software tools allowing the system to be fully managed. Figure 2 shows the defined structure for Cloud Gaming implementation. Both tools run over the thin client. Here, the *actions automation tool* is designed to develop automated testbed configuration and emulate gaming actions. The parameters that can be configured are video resolution, frame rate, audio mode, encoders, and number of iterations for each test. Furthermore, the *Metrics extraction tool* has the function to gather KQI information from each Cloud Gaming session and save it into JSON (JavaScript Object Notation) files.

The connection between the Cloud Gaming server and the thin client is performed by the transport network. In order to establish a group of tests for this service, several technologies were considered over this structure, such as WiFi, Ethernet, and LTE. In the latter case, the element which performs as LTE base station and EPC (Evolved Packet Core) is a CrowdCell, an open-source solution to provide capillary LTE coverage and improve indoor cellular service [42].

The tests are made under a Cloud Gaming server with an 8th generation Intel i7 processor with a dedicated Nvidia RTX 2060 graphics card and network card with Killer controller [43], which is optimized for gaming activity. The client side is comprised of a notebook with 6th generation i5 processor and an integrated graphics card. The hardware limitations in the client are ideal to represent the *thin client* instance needed for a Cloud Gaming paradigm.

On the server side, the Nvidia Experience software is installed allowing to manage the graphics configuration for the host and the game streaming server using the *GameStream* protocol. Besides that, the client side uses the Moonlight-Stream software to configure and manage a Cloud Gaming session with the server. As was previously mentioned, the software tools are implemented and run over the client simultaneously. The following subsections present an overview of each block operation and its functions.

### 3.2. Actions Automation Tool

**Figure 3:**
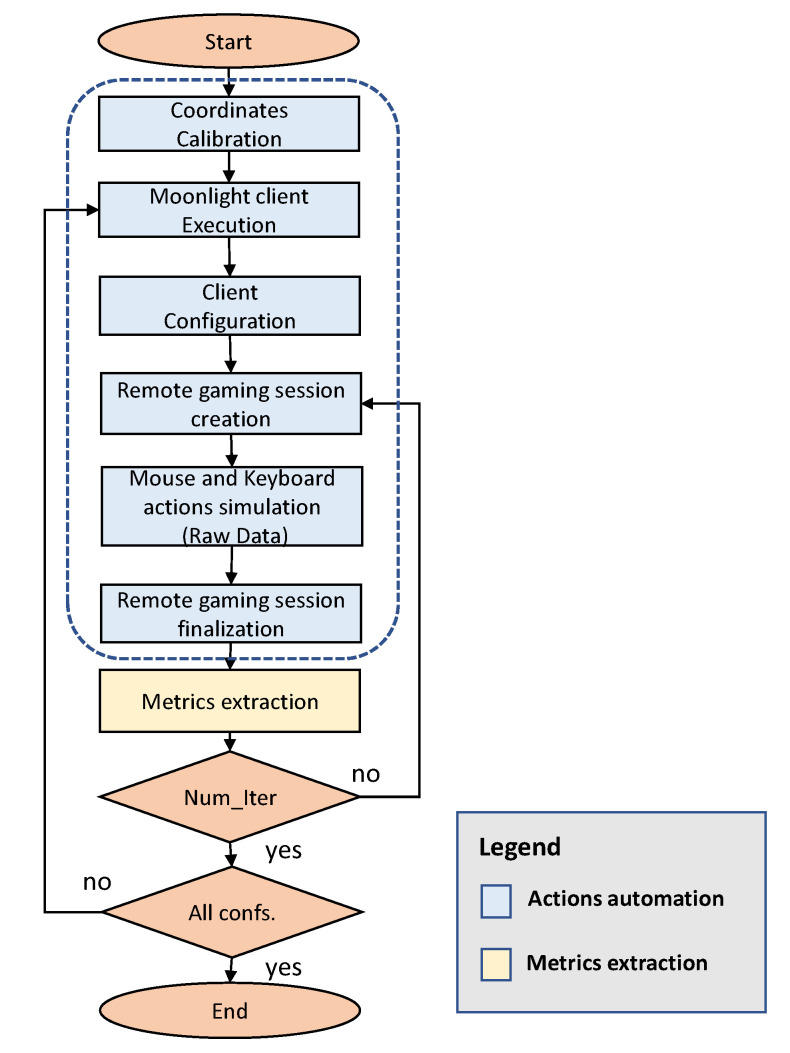
Software tool flowchart.

In order to maintain an objective measurement scheme and the results obtained in the same context, player actions need to be automated. Hence, this block focuses on the automation of several tasks that are necessary to create, manage, control and finish a Cloud Gaming session through the emulation of mouse and keyboard events. Thus, the thin client is able to capture and send the user actions in a compatible way, so the server can interact with the online gaming server, and in this way, emulating the user gaming interactivity. With this goal, the tool performs the following tasks in a sequential scheme, which is shown in Figure 3:

- **Coordinates calibration**: This function configures JSON files that are used for determining X and Y coordinates, for each mouse action simulation during a session. This data is calibrated for several video resolutions in a previous phase before the tests.
- **Client configuration**: This phase is dedicated to set up the session parameters, such as resolution, frame rate, audio mode, coder, and decoder. These elements are configured before a group of tests are launched for the testbed.
- **Moonlight client control**: After the configuration procedure, this block allows the execution, creation, and finalization of a Cloud Gaming session using Moonlight stream client. This function uses actions emulation in the thin client.
- **In-game actions control**: Once the game session is ready, this function emulates the keyboard and mouse actions that are used for simulating the user interaction with game logic. These actions are generated in the thin client, but they are also captured and transported to the server.
- **Metrics extraction**: Finally, this feature permits to manage and launch the metrics extraction tool once the game simulation is finished.

### 3.3. KQIs Extraction Tool

When the Cloud Gaming session has ended, several temporary files are created by the thin client. The extraction tool is designed to search for the suitable file that corresponds to each session. Then, the KQIs are extracted based on timestamps and measurement events. Once the KQI indexing is done, this information is grouped and used for creating CSV (Comma-separated Values) files that save these indicators together with the configuration parameters assumed for each test in the Cloud Gaming system.

**Figure 4:**
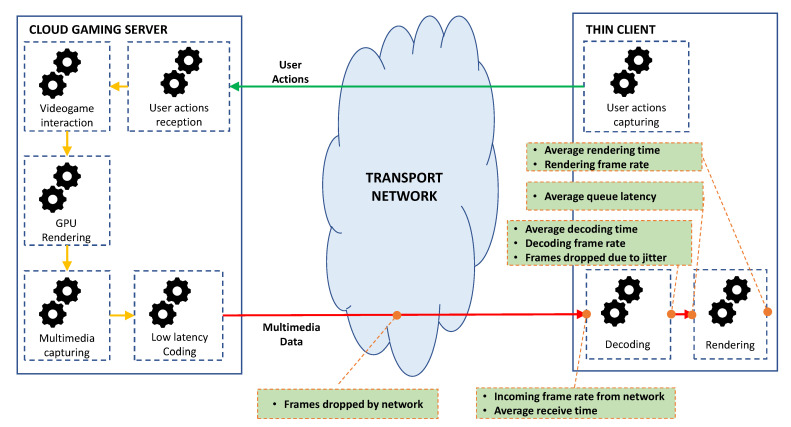
Metrics location in the Cloud Gaming architecture.

Table 2 shows a list of the available KQIs related to a Cloud Gaming service that can be extracted through this tool. Meanwhile, Figure 4 describes the logic location, where each KQI is measured in the context of a gaming session.

The frame rate values are expected to be closer to the configured values in the client (30 and 60 FPS). In general, the principal limitation in the performance of this service is the losses. According to the bibliography, the frame rates should not be lower than 25 FPS. This means that in the 30 FPS case, the maximum admissible losses are about 16%. From this, the expected values for the network losses are set to about 15% and jitter losses to 1% approximate. This assignment considers the network as a bottleneck in the service provision and not as unstable network, which is clearly not recommended for this kind of services.

As mentioned in the bibliography, the gaming experience might be considered normal, not optimal, when the latency does not overpass 100 ms. In this case, as a baseline for our experiment, the rendering time must not be higher than the interframe period, say 1/60 which represents 16.67 ms. Moreover, the maximum time that a frame should await in the queue must not exceed the same period that the rendering stage takes, so the expected value must be about 16.67 ms before the frame expires.

Moreover, the decoding time might be considered to last at least double that of the rendering time (33.33 ms), so the frames have enough time to be decoded considering the time in queue. The average receive time, in the worst case, should consider the difference between the expected latency goal (100 ms) and the sum of the other latencies. This fact gives an estimated value of 33.33 ms.

The information involved in these KQI will allow to find out relations between the parameters that are involved in a Cloud Gaming session. Moreover, this information will help to analyze to what extent the session configuration affects service performance. The evaluation of the proposed framework is presented in the following section, as well as the obtained results, are discussed.

## 4. Evaluation and Discussion

In order to evaluate the developed framework over a real Cloud Gaming system, a testbed was designed and implemented. This is composed of a Cloud Gaming server, a thin client, which has been implemented by a Moonlight Stream client to generate remote gaming sessions, and Python scripts that execute and control the tools. Moreover, to analyze the impact of the network in the system performance, several technologies were considered to be used in the transport particularly LTE, WiFi, and Ethernet.

### 4.1. Setup

**Figure 5:**
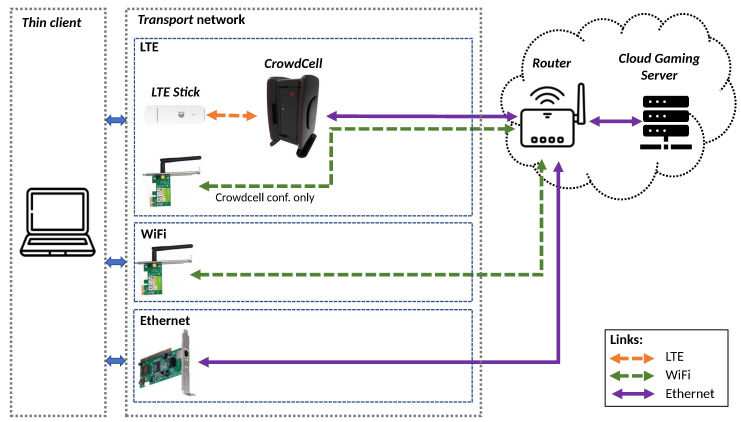
Testbed description.

The testbed is composed of a set of tests that run over the system described in Section 3. In the client, it is possible to configure the video resolution and maximum frame rate with which is streamed from the server. These parameters are configured automatically using the *actions automation tool*. In order to obtain objective results, the testbed is able to generate a specific number of iterations for a fixed configuration. In addition, the testbed allows extending the tests for a combination of resolutions and frame rates for each technology in the transport. The testbed setup is represented in Figure 5.

The Cloud Gaming server is connected via an Ethernet link with a router that provides IP addressing to the network. This network element allows the server to reach the Internet. Otherwise, the transport network is up to link the thin client with the server through several technologies. In Ethernet and WiFi cases, the link is provided by the router and the respective client network cards. In the LTE case, the client uses an LTE Stick (Huawei E3372), which enables to connect to a CrowdCell. This device is able to offer LTE coverage, thanks to the implementation of a virtual EPC by using the Amari 100 LTE [44] software. The procedure to configure the CrowdCell is done using an external WiFi link with the router.

The experiments consider four video resolutions (720p, 1080p, 1440p, and 4K) with two frame rates values (30 and 60 FPS). Audio mode and codec values are set using the default values suggested by Moonlight client. On the one hand, when an Ethernet link is used, the channel bandwidth is determined by the technology (Fast-Ethernet), meanwhile, the WiFi and LTE cases use a fixed 20 MHz channel. In addition, the LTE tests use two different simulated channel conditions for 30 and 10 dB. This feature is evaluated in the CrowdCell side due to the SDR (Software-Defined Radio) capabilities that are available to test in this device.

The data has been extracted using a group of 20 iterations for each combination resolution-frame rate for all the technologies in the transport network. This fact has allowed gathering information related to the impact of the transport network on the service quality. All this information has let the analysis and correlation of the session configuration, including client configuration and video resolution with the estimated KQIs in Cloud Gaming. The values of these parameters have been summarized in Table 3.

### 4.2. Inter-Technology Comparison

According to the previous testbed descriptions for each technology used in the transport network, several experiments have been done in order to establish the impact of configuration parameters in a Cloud Gaming session (such as resolution and frame rate) over the experienced KQIs. In this context, the presented results belong to a 20 MHz bandwidth channel for LTE and WiFi, and Ethernet using a Fast-Ethernet network card. The KQIs that are shown in this paper represent the most important ones in terms of latency and performance: average receive time, average rendering time, percentage of frames that were dropped (by jitter or network), and rendering frame rate at the client (see Table 2).

All the figures that will be presented in this section are divided into two parts. The first one depicts the evolution of each KQI value depending on the experiment. Each one of the technologies used in the transport network is represented using different line styles and colors. The *x*-axis describes the number of the experiment; meanwhile, the *y*-axis stands for the measured KQI value.

The second part shows the configuration for each group of tests. In this context, each group of 20 samples represents a specific configuration. As in the previous part, the *x*-axis represents the number of the experiment; meanwhile, the *y-axis* denotes the video resolution assumed for the groups of tests. Each group of tests that share the same step (40 samples) in the second graph is configured for a specific resolution in the following order: 720p, 1080p, 1440p, and 4K. Furthermore, this graph denotes which tests (20 samples) have been done for 30 FPS (dotted line) and 60 FPS (continuous line).

**Figure 6:**
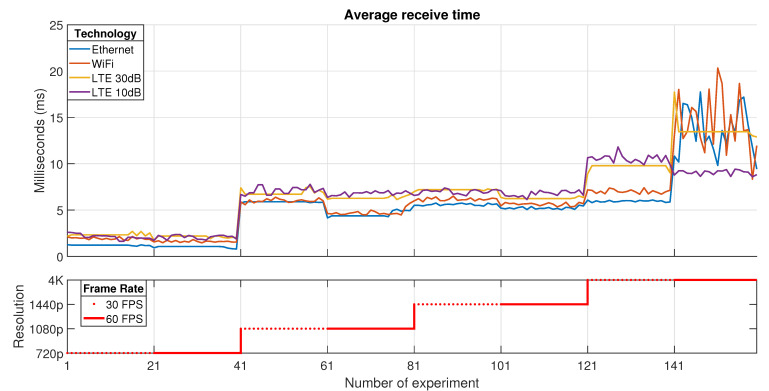
Average receive time.

Figure 6 represents the *Average receive time* in the service. The video resolution is fixed for each group of tests, which means the video is not adaptive in each session. The results show that this latency is highly dependent on the chosen video resolution. This effect is generated due to the increase of data volume (packets) to represent a frame in a higher resolution. For instance, this means that more time is necessary to reassemble a frame in 1080p rather than 720p. In contrast, it is important to note that the use of a higher frame rate (30 to 60 FPS) slightly reduces this latency. When the video frame rate increases, the inter-frame period must be reduced, as a consequence, the server sends frames more quickly to the client in order to guarantee time synchronization. This behavior is compatible with all the test cases until the transport network is unable to handle the traffic (e.g., 4K).

The best times obtained in this work correspond to Ethernet, which use a wired channel instead of LTE and WiFi which are radio technologies. However, when the radio conditions are suitable, LTE shows better performance values than those obtained with WiFi. In addition, it is mandatory to highlight that this metric estimates the average through the non-discarded frames, hence justifying the decrease in LTE 10dB due to frames losses in the network, specifically in 4K with 60 FPS case.

**Figure 7:**
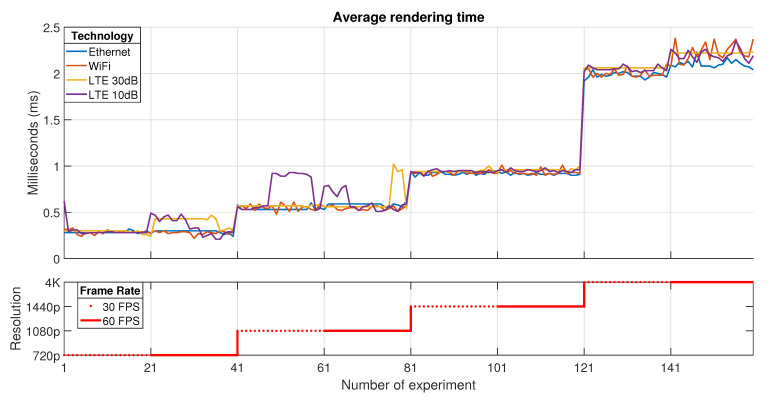
Average rendering time.

The QoE in Cloud Gaming is also affected by processing tasks. In this way, Figure 7 represents the *Average rendering time*. The obtained values indicate that the resolution is the parameter that impacts more in this graphic process. However, the frame rate is a secondary parameter that could reduce this latency in a minor way than resolution. It is important to mention that this KQI depends on the decoding process. If the previous stage takes long, certain frames could be discarded due to lack of time synchronization, hence increasing this processing time. A key point in this KQI is that the transport technology does not impact heavily on the response time because graphic delays clearly depend on thin client hardware capabilities.

**Figure 8:**
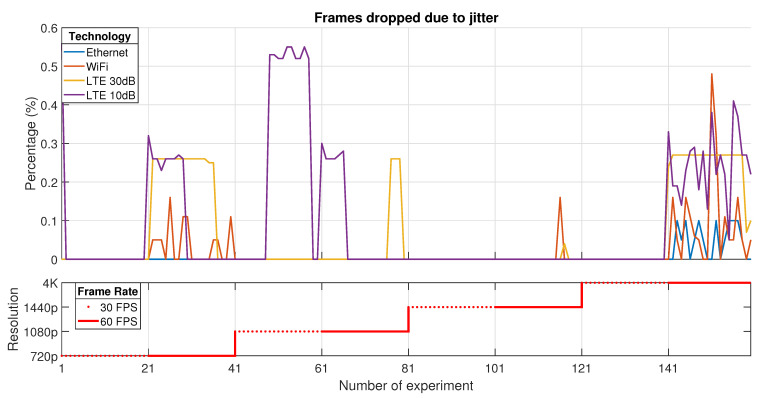
Frames dropped due to jitter.

Conversely, the losses in a Cloud Gaming service are featured by the packet and frames dropped. These losses are due to resource limitations in both the transport network or the client hardware. In first instance, in Figure 8 is represented the percentage of *Frames dropped due to jitter* in the transport and representation of video frames. These effects consider network and decoding delay variations. The first one is network-related due to variations in the usual delivering time. The second one is related to the fact that the client must assure proper decoded frames before sending them to the render. If a frame is received at the client but the decoding process takes longer due to missing or wrong packets, this frame is marked as delayed because of timeout. In both cases, the entire frame is discarded due to the aggregation of an unusual delay that produces considerable jitter in the client, which is not admissible in a real-time service.

As it can be seen, the service presents a better performance over Ethernet, which shows a reduced quantity of lost data as a result of using a wired and direct link between the server and the thin client.

However, for wireless technologies, such as LTE and WiFi, there is an increase in the percentage of losses due to the radio channel. In addition, the LTE case increases the losses related to jitter due to frames that are delayed in buffers, which is a problem in a real-time service. This fact is produced because of the use of the CrowdCell, which introduces additional latency in the transport of the frames. Despite this, the results are equivalent in all the technologies when the tests are configured for the best quality, that is to say, 4K resolution with 60 FPS (<0.6%).

**Figure 9:**
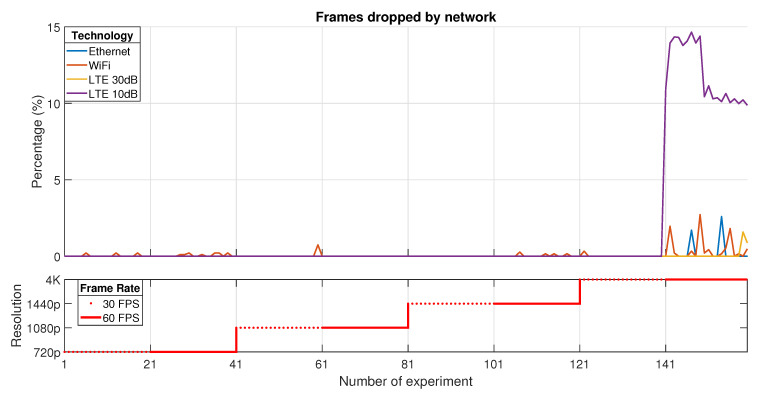
Frames dropped by network.

The losses, which are generated due to different effects than jitter, are directly related to network capabilities in order to transport all the multimedia data. In this case, Figure 9 indicates the percentage of *Frames dropped by the network*. This kind of loss is produced by two different reasons that affect the performance in the service. On the one side, the frames that were not able to be reassembled in the client due to timeout must be discarded before using computational resources in the decoding process. On the other side, there are certain cases when the frames are reassembled but they took more time than an inter-frame period to be ready for the decoding process, this way, these frames are expired and providing decoding resources is not suitable.

As it seems, the results are less dispersed than in the previous KQI. Nevertheless, this indicator presents its maximum values whereas the 4K resolution with 60 FPS rate is used.

It is possible to recognize that Ethernet produces the lowest frame dropping percentages. This confirms that a wired medium is always the best choice for real-time services. However, when LTE channel has suitable conditions, the losses are about 3% in the worst case. This behavior is not similar when the channel is not adequate as in LTE 10 dB case. Taking account into the results, using LTE as the transport technology could provide improved performance than WiFi, even including the fact that mobility could be included as an extra feature in the service provision.

**Figure 10:**
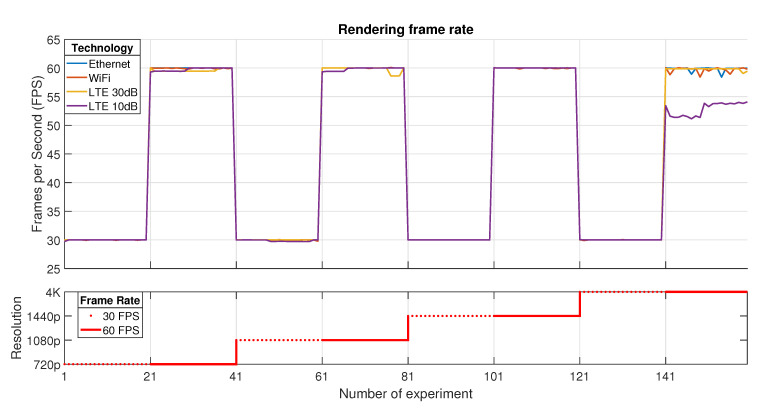
Rendering frame rate.

The last KQI presented in this article is referred to the performance of a Cloud Gaming service. Figure 10 represents the *Rendering frame rate*, which is obtained as a consequence of the previously decoded frames and takes into account all the losses in the transport of the multimedia streams. Gathered results show that the overall system works perfectly for a 30 FPS rate no matter which either resolution or technology is used.

To contrast this context, when a 60 FPS session is configured, the system slightly reduces the rendering frame rate in all cases due to the hard-graphic processing effort that the client needs to do. The most relevant case occurs when 4K is used with a 60 FPS rate, where all the mentioned frame losses represented in Figure 8 and Figure 9 affect the decoding and later rendering process, reducing the QoE of the service.

### 4.3. KQIs and Configuration Parameters Relationships

The previous analysis was made in order to describe the behavior of each metric with different technologies in the transport network. In this section, the overall relationships that each configuration parameter has with each KQI is presented in order to measure its grade of impact. To do this, a correlation matrix is calculated to describe the magnitude of dependency for each configuration parameter over the system response. Values that are close to 1 means maximum relation; meanwhile, values closer to 0 represents that there is not any relationship between those parameters.

It is important to mark that all the experiments for this specific analysis have been done using LTE as technology in the transport network. The gathered data was extracted under the configuration that is described in Table 3.

**Figure 11:**
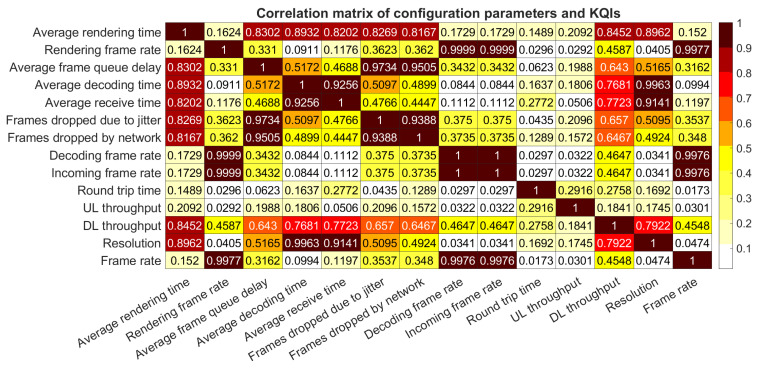
Correlation Matrix of Configuration parameters and KQIs.

Figure 11 shows that all the KQIs which represent frame rates are correlated with each other. That proves that the *Rendering frame rate*, as the last stage in the service, is a consequence of previous phases that involves receiving frames from the network and decoding them in the thin client. In the same context, all processing latencies are related among themselves, which means that a delay in the frame reception process corresponds to a delay in the rendering phase.

In addition, packet losses due to both reasons (jitter and network) are correlated with high latencies in the service. As a consequence, if the decoding process gets delayed because of corrupted or expired frames, the rendering does, as well. In this context, it is possible to affirm that frame losses can affect the overall system.

To support this analysis, it is necessary to identify how input parameters, such as video resolution, frame rate, or bandwidth, affect the gathered KQIs. So, it is possible to see in Figure 11 that the video resolution has a high impact on the system’s delays, such as average receive time, decoding time, rendering time, and so on. In the same way, this parameter is highly related to the frame losing (jitter and network). This point matches with the previous relation analysis between KQIs.

Moreover, the frame rate is correlated with the graphic performance of the system. As this rate increases, the need for decoding and rendering raises in the thin client. In some cases, when a high frame rate is combined with a high resolution, it could impact negatively on the overall performance because of the decreasing frame rate due to lack of either arrived frames or processing capacity.

The last parameter that can be analyzed is the bandwidth. The obtained results show that the bandwidth represented by throughput in both channels, uplink and downlink, is an important factor that must be taken into account for optimization analysis. As it is possible to see, the correlation matrix indicates that the downlink throughput impacts heavily over most parameters. A reduced bitrate in downlink may produce bottlenecks in the transport network, hence delaying frames, adding latencies in decoding, rendering processes, and, in the worst case, losing crucial information. Otherwise, the uplink channel does not require large bitrate requirements, this way, it has no impact over the network. Nonetheless, this information must be guaranteed in order to assure responsiveness in Cloud Gaming.

**Figure 12:**
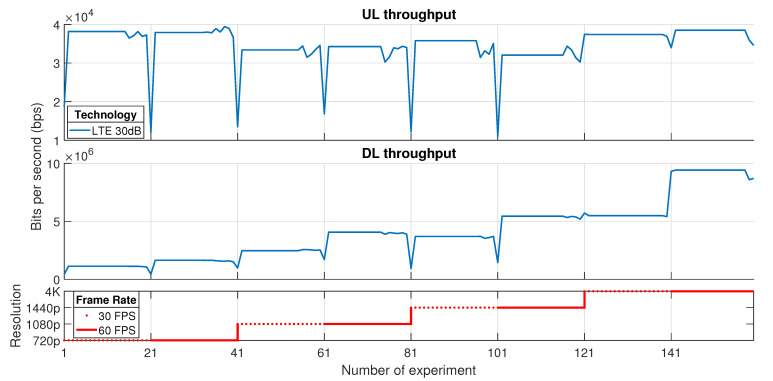
Uplink (UL) and Downlink (DL) bitrate in Long Term Evolution (LTE) testbed.

Figure 11 has shown there is a relation between the downlink channel, the bandwidth and the session configuration. To support this fact, it is necessary to analyze the throughput evolution depending on the bitrate and the proposed configurations for resolution and frame rate. The results obtained in this specific experiment are presented in Figure 12. The *x*-axis represents the number of the experiment; meanwhile, the *y*-axis, in both graphs, depicts the value of throughput, which is measured using the CrowdCell, for uplink and downlink channel, respectively. In addition, the third graph shows the configuration for each experiment. The resolution and frame rate parameters are depicted with different stairs and line styles; however, each test considers 20 iterations for a specific configuration.

The results in Figure 12 shows, on the one hand, that the downlink bitrate increases as the resolution does. The frame rate affects slightly on the raise of throughput in this direction. Conversely, in the uplink channel, there is a flat relation amongst the bitrate and the assigned combination between resolution and frame rate. The UL (Uplink) bitrate is featured by metadata which contains user actions plus client data. These facts assure the critical volume of traffic is presented just in DL (Downlink) direction. However, both data flows must meet specific treatment in the network due to its time-sensitive nature. If the bitrate is hardly limited in uplink, the packets take more time to be delivered to the server, which means losing responsiveness due to high end-to-end latency and, in the worst case, packet dropping and session interruption. On the other side, an increase in DL traffic and abounded channel bandwidth produces bottlenecks and consequent multimedia packet losses. The lack of this information on both sides could produce QoE degradation in Cloud Gaming.

**Figure 13:**
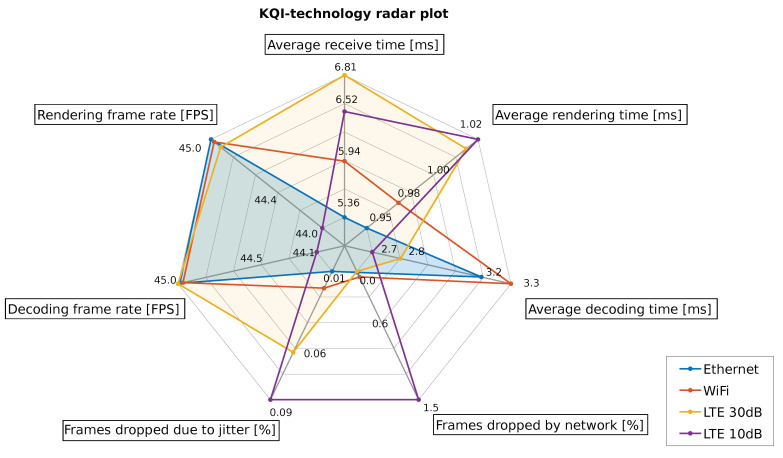
KQI performance for the assessed technologies.

In order to understand how the assessed network technologies have overall performed the Cloud Gaming testbed, a radar chart was designed. Figure 13 shows a graphical and qualitative description of the average KQI values for each technology. The value labels have been hidden, and the KQI axis scales have been adjusted for each KQI, to assure a clear understanding and maximize the difference between the technologies.

As it is possible to see, Ethernet is the technology that provides the best values in terms of high frame rates, low latencies in frame transport and graphics processing, and low frame losses. From this, WiFi presents high frame rate values but lower than Ethernet, and the latencies are higher. Moreover, the average decoding time is higher than Ethernet due to losses because of network and jitter issues, which delay the decoding process in the client.

On the other side, LTE presents two different situations. Firstly, when the channel conditions are not enough good, the frame losses due to jitter and network are quite higher than Ethernet. This fact increases the average receive time and reduces the average time of the decoding process. This last one case is because the calculations consider only the healthy frames which arrived at the client to estimate the average decoding time. However, an increase in the rendering time is expected due to the lack of certain frames. The main consequence of losses in this service is the reduction of the frame rate and the consequent reduction in the video smoothness on the client-side.

Secondly, when the LTE channel presents good conditions the losses are quite reduced. Figure 13 shows that LTE performs lower losses due to network in comparison with WiFi. In contrast, the losses because of jitter are slightly higher. It is important to remark that this last KQI scale is about 0.3%, which means almost negligible. In addition, the increase in the average receive time calculation respect WiFi is due to the augmentation of the number of healthy frames which are delivered to the client. Moreover, the lower losses in the service, the better frame rate is presented to the user, so gaining video smoothness.

To summarize, Ethernet provides the best performance for Cloud Gaming provision due to its wired nature. In contrast, WiFi and LTE are wireless technologies that perform slightly lower than Ethernet. Nevertheless, LTE presents lower losses due to the network than WiFi, providing the chance to handle improved frame rates with reduced losses and consequently rising the QoE.

This section has presented an integral analysis of the relations between the Cloud Gaming session configurations represented by the parameters resolution and frame rate and KQIs values. The results have shown that as requirements increase, the service QoE can be compromised. In general, if the resolution is augmented, the downlink traffic produces bottlenecks that cause losses and increasing latencies. In the same context, an increment in the frame rate produces QoE degradation related to major computational costs; however, its impact is lower than the produced by resolution changes.

In the same context, an increment in the frame rate produces QoE degradation related to major computational costs; however, its impact is lower than the produced by resolution changes.

Additionally, the correlation matrix in Figure 11 has demonstrated that the configured bitrate can influence over the system responsiveness. This indicates that a wider session bandwidth can improve the traffic delivery on both sides of the communication. In this service, both uplink and downlink data are important to assure a precise gaming experience with reduced latencies and packet loss issues.

Finally, the radar chart qualitative analysis has demonstrated the utility of LTE in the Cloud Gaming provision. The facts have shown there is improved performance when LTE is used in the testbed. This means that this technology has less percentage of losses, higher frame rates in the client and similar computational latencies in comparison with WiFi taking into account Ethernet as the baseline.

## 5. Conclusions & Outlook

The popularization of services managed in the cloud has achieved levels with no precedents due to the evolution of networks and their services-based architectures. The Cloud Gaming approach is one of these cutting-edge services which try to offer the users the ease to enjoy a gaming experience without the need to own graphics-dedicated hardware at a high cost. To reach this, all the computing tasks are developed by servers, then video streaming-based content is sent to the user. This last one feedbacks the server with its actions to interact with the game.

As is expected, this service faces some specific network restrictions that are completely different from a traditional video streaming service. Cloud Gaming needs reduced latency on both sides of the communication to assure adequate responsiveness. However, this is not the only challenge that must be confronted. The video in the downlink must assure the video quality in terms of resolution and frame rate to reach a suitable smoothness.

These facts led to some key points that should be faced to improve the QoE in this service. In this context, the present work has established a framework to experimentally assess the performance in terms of session configuration parameters, such as resolution and frame rate, and the network impact. This has been implemented in a real testbed to assess this service under different communication technologies (Ethernet, WiFi, and LTE technology) and configurations.

On the one hand, the gathered data have let an analysis on the behavior of Cloud Gaming metrics. Remarkably, losses on both sides of the communication can negatively affect service provision on different scales. In the downlink, the loss of frames due to network effects as system jitter increases the processing latencies that involve decoding, queuing, and rendering stages. In addition, the frame dropping in the client’s screen is a consequence of an increment in delays which means that certain frames are completed when they are expired or not suitable to be represented on screen. This is due to the real-time nature of this service, which requires smoothness, this way causing video tearing and poor quality that, in the worst case, the possible reduction in willingness to play.

Moreover, on the uplink side, the loss of data, which contains session information and the user actions, might produce a lack of control in the gaming environment. The consequence of this failure reduces the required responsiveness, which is mandatory in this kind of service, where the user notices a change in milliseconds accord to the bibliography. Both downlink and uplink case disturbances reduce drastically the quality of the experience in Cloud Gaming.

On the other hand, the obtained results show the performance of each tested technology. All of them respond well when the resolution and frame rates are manageable, in terms of computational resources and traffic volume. As the video quality increases, mostly characterized by the resolution, the wireless technologies present frame losses that reduce frame rate on the user side. However, it is remarkable that LTE and WiFi perform quite better as expected when the resolution is fixed to 4K and 60 FPS in the frame rate. Besides, LTE exhibit overall fewer losses than WiFi, which means more suitable video quality and smoothness (higher frames rates), and responsiveness, even in the most demanding case.

Summarizing, the qualitative as quantitative analysis has demonstrated that the wireless technologies might be adequate to provide Cloud Gaming service as an alternative to the traditional wired ones. Nevertheless, service-specific network optimization is recommended to assure the best quality of experience possible.

Novel research challenges that arise from this work are, on the one hand, the optimization of the Cloud Gaming platforms, to reduce the impact on the QoE of limited computational or communication resources. On the other hand, network optimization might be done by applying Machine Learning approaches to face the service requirements in terms of packet budget, priority, minimum guaranteed throughputs in the RAN as the policies in the backbone for packet handling.

Besides, future work is planned to extend the analysis to additional Cloud Gaming existing platforms, as well as to 5G scenarios, where the use of novel technologies, such as network slicing, are expected to support the fine-tuning of resources and configuration to fully optimize the Cloud Gaming experience.

## Figures and Tables

**Table 1:** Cloud Gaming open-source platform comparison.

Feature	Gaming Anywhere [30]	Rainway [31]	Moonlight [32]
Server-Client Architecture	Yes	Yes	Yes
Multimedia stream transport protocol	RTP	WebRTC	RTP
User actions transport protocol	TCP	WebRTC	UDP
Version control	Discontinued	GitHub	GitHub
Server host operating system	Linux	Windows	Windows
Graphics card requirement	O.S. Compatible	Agnostic	Nvidia GTX/RTX
Client host operating system	Multi-platform	Multi-platform	Multi-platform
Client configuration	Script	Interface	Interface
Game implementation	Manual	Search engine (MIST)	Search engine (Nvidia Experience)

**Table 2:** Available Key Quality Indicators (KQIs) for each Cloud Gaming session.

Parameter	Description	Expected Value
Incoming frame rate from Network	Average estimated number of frames that are received in the thin client network interface.	Maximum as configured (30 or 60 FPS)
Decoding frame rate	Average number of decoded frames in the client.	Maximum as configured (30 or 60 FPS)
Rendering frame rate	Average number of rendered frames in the client.	Maximum as configured (30 or 60 FPS)
Frames dropped by network	Average percentage of lost frames in the transport process due to network errors or hardware limitations.	<15%
Frames dropped due to jitter	Average percentage of lost frames in the client buffer due to jitter.	<1%
Average receive time	Average time that an encoded frame needs to be completed since the first packet was sent from the server. The time estimation is just done among the non-discarded frames	<33.33 ms
Average decoding time	Average time that a reassembled frame needs to be decoded in the client.	<33.33 ms
Average rendering time	Average time that a decoded frame needs to be rendered and represented in client’s screen. This KQI consider V-SYNC latency.	<16.67 ms
Average frame queue delay	Average time that a decoded frame waits in the queue before the rendering process.	<16.67 ms

**Table 3:** Setup configuration.

Parameter	Configuration
Resolution	720p, 1080p, 1440p and 4k
Frames per second (FPS)	30 FPS and 60 FPS
Audio mode	Stereo
Video decoding	Software
Video encoder	H.264
Number of iterations per configuration	20
Ethernet bandwidth	100 Mbps
WiFi Bandwidth channel	20 MHz
LTE Bandwidth channel	20 MHz
LTE Band	7
LTE Duplexation mode	FDD (Frequency Division Duplex)
LTE CrowdCell SNR (Signal-to-Noise Ratio)	10 and 30 dB

## Data Availability

Not applicable.
